# YSK_2_ Type Dehydrin (*SbDhn1*) from *Sorghum bicolor* Showed Improved Protection under High Temperature and Osmotic Stress Condition

**DOI:** 10.3389/fpls.2017.00918

**Published:** 2017-05-30

**Authors:** Tanmoy Halder, Gouranga Upadhyaya, Sudipta Ray

**Affiliations:** Plant Functional Genomics Laboratory, Centre of Advanced Study, Department of Botany, University of CalcuttaKolkata, India

**Keywords:** dehydrin, lactate dehydrogenase, protein aggregation, high temperature stress, osmotic stress

## Abstract

YSK2 type dehydrin from *Sorghum bicolor* (SbDhn1) showed a high level of transcript accumulation when subjected to high temperature and osmotic stress. The high transcript level occurring in such stress situation might lead to a protective effect; though the exact mechanism by which this is achieved remains poorly understood. Nevertheless, our results provide compelling evidence to prove that transgenic tobacco lines overexpressing *SbDhn1* gene showed improve stress tolerance as assessed by reduced membrane damage and low MDA content. Furthermore, we demonstrate here *SbDhn1* expressing lines were only able to recover after stress treatment. In this study, we have provided direct evidence for the protection rendered by SbDHN1 protein to a temperature-sensitive enzyme under both high temperature and osmotic stress. We extended this analysis to the whole plant proteome where the addition of SbDHN1 protein helped in retaining the solubility of the protein was demonstrated. Interestingly, *in vitro* experiments carried out with lactate dehydrogenase (LDH), showed aggregate formation upon subjecting it to high temperature. However, in presence of SbDHN1 protein very few aggregates were observed. Aggregation assay showed a high level of aggregates in wild-type or empty vector transformed plants as compared to *SbDhn1* transgenic lines. Confocal microscopy images in leaf peel sections of wild-type plants showed high amounts of aggregates as compared with transgenic lines. This study provides evidence for the protection rendered by SbDHN1 protein under high temperature by inhibiting the aggregate formation and provide the rational for the mechanism how these proteins ameliorate the adverse stress conditions.

## Introduction

Plants face several environmental challenges during their life cycle. A complex mechanism exists in plants which help them to sense, react, and adapt accordingly. Several genes were shown to be up-regulated by abiotic stresses such as cold, drought, or salt stress. Plants express several stress response proteins, like the LEA (late embryogenesis abundant) protein as a response to osmotic stresses (Close, 1997; Allagulova et al., 2003; Rorat, 2006; Kosová et al., 2007; Graether and Boddington, 2014). The LEA proteins are ubiquitously distributed across the biological kingdoms from prokaryotes to eukaryotes (Tunnacliffe and Wise, 2007). These proteins were first characterized from cotton and wheat (Cuming, 1999) and were abundantly found during development of seeds. The LEA proteins comprise about 4% of the total cellular proteins (Roberts et al., 1993). The expression of these proteins is coupled with the acquisition of osmotic stress tolerance in orthodox seeds, pollens, and anhydrobiotic plants. The precise function of these proteins is yet unknown, but are assumed to protect the cellular or molecular structures from the damaging effects of environmental stresses. The LEA proteins can be grouped into eight families in the PFAM database (Finn et al., 2010) according to their primary sequences: dehydrin, LEA_1, LEA_2, LEA_3, LEA_4, LEA_5, LEA_6, and seed maturation protein (SMP). The LEA proteins (PFAM: LEA_4; LEA_5 and dehydrins) are endowed with protective mechanisms to circumvent the structural changes in the membranes and biological macromolecules associated with osmotic stress tolerance. The induced expression of these proteins in seeds might be one component by which the seeds reach a condition of ‘suspended animation’ and yet remain viable. However, the stress assault might induce misfolding and aggregation of proteins (Ben-Zvi and Goloubinoff, 2001; Zhou et al., 2008). The misfolded and aggregated proteins might lead to damaging consequences for the cell (Bucciantini et al., 2002). Prevention of conformational changes in the biological macromolecules by these LEA proteins might aid in achieving osmotic stress tolerance in plants. The enzyme citrate synthase, which is susceptible to aggregation at high temperature, was used for studying heat-induced aggregation. Recombinant forms of AavLEA1, a group 3 LEA protein from *Aphelenchus avenae*; and Em, a group 1 LEA protein from wheat, can independently protect citrate synthase from aggregation due to osmotic and freezing stress. Similar results were also obtained for the enzyme lactate dehydrogenase (LDH). Heat stress experiments carried out with citrate synthase exhibited a protective effect synergistically with a chemical chaperone trehalose (Goyal et al., 2005). LEA proteins from different groups: dehydrins DSP16 and ERD10 (group 2), AtLEA76 (group 3), AtD113 (group 4) were efficient protectants of enzyme activities during *in vitro* dehydration assay (Reyes et al., 2005). The AavLEA1 from *A. avenae* showed anti-aggregation activity (Chakrabortee et al., 2007). MtPM25, a member of the group 5 LEA proteins was found to protect the whole proteome of *Medicago truncatula* seeds under osmotic stress (Boucher et al., 2010). The MtPM25 was found to prevent aggregate formation after freeze-thawing as visible from micrographic studies. Dehydrins like ERD10 and ERD14 can act as potent chaperones under *in vitro* conditions. ERD10 and ERD14 showed that these proteins were able to prevent heat-induced aggregation and or inactivation of various substrates. Previous experiments carried out with ERD10 and ERD14 showed that the proteins were also capable of binding to the membrane. The binding was influenced strongly by the ionic strength. ERD10 and ERD14 have a chaperonic activity of rather wide substrate specificity (Kovacs et al., 2008). However, the exact mechanism by which these proteins render protection still remains an enigma. Dehydrins, a LEA protein, accumulate abundantly as a stress-responsive protein. Dehydrins are known to be rich in glycine, highly hydrophilic proteins, with intrinsically disordered structure in the hydrated state. Dehydrins render protection from various abiotic stresses like water, salt, temperature, and oxidative stress. Dehydrins have been shown to act in sequestering ions, stabilizing membranes, or acting as chaperones in earlier studies (Rorat, 2006; Tunnacliffe and Wise, 2007). Several reports have also been documented for the protection rendered by dehydrins under freezing stress. Dehydrins from several plant species were revealed to possess cryoprotective activity (Hara et al., 2001).

According to the Close (1997), all dehydrins possess a conserved K-segment (EKKGIMDKIKEKLPG) usually present near the C-terminus. Dehydrins may also possess three other segments: a track of Ser residues (the S-segment), a consensus motif, T/VDEYGNP (the Y-segment), found near the N-terminus; and a random Φ-segment (Close, 1997). Based on the distribution of these segments dehydrins are grouped into five subclasses namely Y_n_SK_n_, Y_n_K_n_, SK_n_, K_n_, and K_n_S.

In this study, we report the occurrence of a YSK_2_ type dehydrin (SbDHN1) in *Sorghum bicolor* which gets up-regulated several folds in *S. bicolor* under high-temperature stress condition. Our experiments demonstrate that the transgenic tobacco plants overexpressing the *SbDhn1* gene could survive high temperature and osmotic stress when fitted in a diurnal pattern of stress treatment for 15 days. To the best of our knowledge, no other genes have been shown to render protection in such a specific stress regime. The heat shock proteins when overexpressed, were capable of protecting the plants, but only for a limited time period (Grover et al., 2013). Unifying all the evidence from our *in vitro* and *in vivo* experiments we conclude that SbDHN1 was able to confer protection to the plant proteome when subjected to high temperature and osmotic stress, a condition mimicked for drought stress. Whether this protection is brought about by dehydrin acting as a molecular chaperone with wide substrate specificity as proposed by Kovacs et al. (2008) or by anti-aggregative properties which may simply be a space filling activity that prevents those interactions leading to protein aggregation (Chakrabortee et al., 2007; Tunnacliffe and Wise, 2007) remains to be worked out.

## Materials and Methods

### Isolation and Sequence Analysis of SbDhn1

Total RNA was isolated from Sorghum plants cultivar B-35 (genotype BTx642) using the RNeasy plant mini kit (Qiagen, Germany). cDNA was prepared using RevertAid First strand cDNA synthesizing kit [Thermo Scientific, (EU) Lithuania] according to the manufacturer’s instruction, using oligo dT primer. The full-length open reading frame (ORF) of SbDhn1 was PCR-amplified using gene-specific primers (Supplementary Table S1) designed according to *S. bicolor* full-length cDNA sequence. The amplified PCR product was cloned into pGEMT-Easy vector (Promega, United States) and subsequently sequenced. The sequence obtained was virtually translated and used for BLAST analysis (Altschul et al., 1990) with the available report in the database (Accession no. AGS16688.1). All of the physicochemical parameters were calculated using ExPASy ProtParam tools. The virtually translated sequence of SbDHN1 was used to compare the protein with other dehydrin sequences from different plant species belonging to the family Poaceae. The alignment was performed using Clustal Omega^1^ program. A phylogeny was generated using dehydrin sequences from different members of Poaceae, belonging to different dehydrin types, using the Neighbor-Joining method in MEGA6 (Tamura et al., 2013). A bootstrap value was obtained from 1000 replicates.

### Stress Treatment for SbDhn1 Expression Study

Sorghum seeds after sterilizing were germinated on half-strength MS (Murashige and Skoog, 1962) medium. Ten days old seedlings were used for the stress treatment. Four different sets each containing four seedlings were taken for the stress treatment. Three sets were placed under different stress conditions like high temperature (48°C), low temperature (4°C), and osmotic stress conditions (MS medium supplemented with 200 mM sorbitol) where as the fourth set was kept under normal cultured condition (unstressed). Samples were collected from each set at different time point 0, 12, 24, and 48 h. Total RNA was isolated using RNeasy plant mini kit (Qiagen, Germany) and cDNA was prepared according to manufacturer’s instruction.

### qRT-PCR for SbDhn1 Expression Analysis

The expression of *SbDhn1* in sorghum under different stress treatment was analyzed by quantitative real-time PCR (qRT-PCR). The EIF1 gene was used as an internal control to normalize the sample amounts. All primers were designed by Primer3 software. The primer sequences used for the real-time experiment are listed in Supplementary Table S1. The qRT-PCR was carried out using the Step One plus (ABI, United States) and SYBR Green Premix (ABI, United States). The PCR cycling conditions were as follows: 95°C for 2 min, followed by 40 cycles of 95°C for 10 s and 55°C for 20 s. A melting curve analysis was routinely performed after 40 cycles to verify primer specificity. The relative expression level was calculated using the 2^-ΔΔCT^ method, in which CT indicates cycle threshold. ΔΔCT = (CT_SbDhn1(stressed)_ - CT_EIF1(stressed)_)_TimeX_ - (CT_SbDhn1(unstressed)_ - CT_EIF1(unstressed)_)_TimeX_. Where Time X is any time point (0, 12, 24, or 48 h).

### Generation of *SbDhn1* Overexpressing Tobacco Line

The SbDhn1 gene was subcloned into pENTR1A vector at *Eco*RI and *Xho*I site followed by subcloning in gateway destination vector pGWB14 under CaMV35S promoter and NOS terminator along with an HA tag; using LR clonase (Invitrogen, United States) according to manufacturer’s instruction. The positive clone was subsequently mobilized into *Agrobacterium tumefaciens* (LBA 4404) using the freeze-thaw method.

Tobacco leaves (*Nicotiana tabacum*; variety SR) were transformed according to Horsch et al. (1985). Green healthy mature leaves were taken from 1-month-old sterilized plants of *N. tabacum*. The leaf lamina was cut into small pieces and pre-cultured in regeneration medium (MS medium supplemented with BAP 2 mg/l and NAA 0.2 mg/l) at 26°C under continuous illumination for 2 days. The Agrobacterium culture was grown in LB medium supplemented with kanamycin and rifampicin at 50 mg/l at 28°C and shaking at 180 rpm for overnight. The overnight grown culture was centrifuged at 6000 rpm for 10 min at 4°C; the pellet was washed with 10 mM MgSO4 and finally resuspended in 10 mM MgSO4. The O.D. was adjusted to 0.9–1.0 at 600 nm by diluting with liquid MS medium. The precultured leaf disks were immersed in the bacterial suspension for 30 min in dark and then transferred to the regeneration medium. The leaf disks were maintained in plant growth chambers for 2 days under the dark condition and then transferred to the regeneration medium (MS medium supplemented with BAP 2 mg/l and NAA 0.2 mg/l) supplemented with 250 mg/l cefotaxime and 20 mg/l hygromycin for embryogenic calli induction; and maintained at 26°C under continuous illumination. Subculturing was done at an interval of 7 days till generation of small shootlets; the small shootlets were placed in basal MS medium (without any growth regulator) supplemented with 250 mg/l cefotaxime and 20 mg/l hygromycin for root induction. After root induction the putative transgenic plants were grown in basal MS medium supplemented with 20 μg/ml hygromycin. The plants regenerated from *in vitro* transformation were considered as T_0_ plants (heterozygous for the insertion). T_1_ transgenic lines were generated by selfing those T_0_ plants and all the seeds were germinated in MS medium supplemented with 20 mg/l hygromycin. The transgenic plants were confirmed by PCR amplification of *SbDhn1* and hygromycin gene (*hpt*) from genomic DNA and cDNA. Though at that stage it was not clear whether those are homozygous or heterozygous for the transgene. Genomic DNA was isolated from the young leaves according to Dellaporta et al. (1983). The isolated DNA was used as a template for PCR amplification. Total RNA isolation and cDNA preparation were carried out as mentioned earlier. Details of the oligonucleotide primers used for amplification of SbDhn1 and hpt genes are given in Supplementary Table S1. Upon expression, in transgenic lines, the HA-tagged SbDHN1 protein was checked by immunoblotting using anti-HA antibody. Total protein was isolated from 1-month-old transgenic tobacco lines along with the empty vector transformed line and wild-type plants. Briefly, 100 mg leaf tissue was homogenized in 1 ml of protein isolation buffer (50 mM Tris-HCl, 100 mM NaCl, 1 mM DTT, 15 mM EGTA, 0.2% Triton X-100, 10% glycerol, 1 mM PMSF) in ice. After centrifugation at 3000 rpm for 10 min at 4°C; the supernatant was collected in a fresh tube and centrifuged at 10,000 rpm for 10 min at 4°C. The supernatant was collected and used for immunoblot analysis after measuring the protein concentration by Bradford method.

For immunodetection of the expressed protein, 5 μg of the isolated protein from tobacco leaves were separated in 12% SDS–PAGE and blotted onto PVDF membrane for 1 h at 300 V. After transfer, the membrane was blocked using 3% BSA solution for 2 h. The membrane was washed with 20 ml TBS-T (20 mM Tris pH7.5, 150 mM NaCl, 0.1% Tween-20) for three times; the blots were then probed with rabbit anti-HA primary antibody (GeNie, India) (1:3000 dilution in TBS-T), at 4°C for overnight with constant shaking. The membrane was washed with 20 ml TBS-T five times to remove the unbound primary antibody and incubated with goat anti-rabbit IgG HRP conjugated secondary antibody (GeNie, India) (1:3000 dilution in TBS-T) for 1 h at 37°C. The membrane was washed and incubated with Luminol western blotting substrate (Thermofisher) according to the manufacturer’s instruction. The membrane was wrapped in a zip seal bag and exposed to Kodak [XBT] film for 10 s to 1 min. The film was developed, followed by fixation and finally washed with water and air-dried.

### Abiotic Stress Treatments

The T1 transgenic lines and empty vector transformed lines along with wild-type tobacco plants; grown under tissue culture conditions were transferred to MS agar supplemented with 200 mM sorbitol and grown in growth chamber in 3000 lux white light and 16/8 h photoperiod for 21 days in order to evaluate the extent of tolerance rendered by introgression of *SbDhn1* gene in tobacco plants.

A combination of osmotic stress along with a variation of temperature ranging from 32 to 48°C was used in order to mimic the exact drought situation as encountered by plants in drought-prone areas of the world. T1 transgenic tobacco plants harboring *SbDhn1* gene (Lines 1, 6, and 10), empty vector transformed line and wild-type plants, were grown in soilrite for 21 days. Water was withheld for 7 days before imparting temperature stress and continued for another 14 days at a high-temperature stress regime as shown in Supplementary Figure S7.

In both the cases plants were photographed before and after stress treatment. The chlorophyll content, soluble sugar content, relative electrolyte leakage and malonyl dialdehyde (MDA) content were estimated in order to evaluate the physiological condition of the plants after stress treatment. The wild-type plants without any stress treatment served as a control in this experiment. All the experiments were repeated at least three times and the results obtained from the transgenic lines were compared with the wild-type plants in order to calculate the statistical significances of the data; *p*-values were calculated using student’s *t*-test. ±SEM was calculated from at least three replicates.

#### Estimation of Chlorophyll Content

The chlorophyll content in the leaves was measured spectrophotometrically after extraction of 50 mg leaf tissue in 5 ml 80% acetone according to Arnon (1949). The total chlorophyll content was calculated using the formula given below:

$$\begin{array}{c} \mathrm{Total}\mathrm{chlorophyll}=\frac{[20.2(D645)+8.02(D663)]V}{(1000)W} \end{array}$$

Where D, optical density value; V, final volume of 80% alkaline acetone; W, fresh weight in g of tissue extract. The total chlorophyll content was estimated in the term of mg of chlorophyll/g of fresh tissue.

#### Estimation of Soluble Sugar Content

Total soluble sugar content was analyzed using the method described by Irigoyen et al. (1992). Total soluble sugar was determined using glucose as standard and expressed as mg/g dry weight of leaves. Approximately 100 mg of ground leaf sample was homogenized in 1 ml 95% ethanol and centrifuged at 11,000 rpm for 10 min. one hundred microliter supernatant was mixed with 3 ml of anthrone solution [150 mg anthrone in 100 ml of 72% H2SO4 (w/w)] and then incubated in boiling water for 10 min. After cooling, the light absorptions of the samples were estimated at A625 using Jasco V730 IRM spectrophotometer.

#### Relative Electrolyte Leakage Estimation

Relative electrolyte leakage was used to evaluate the cell membrane damage after stress treatment. The leaves were excised from the wild-type, empty vector transformed and *SbDhn1* transformed plants subjected to stress treatment for determining the relative electrolytic leakage. The wild-type plants not exposed to any stress served as a control in this experiment. The leaves were rinsed and incubated in 20 ml of deionized water and kept in the dark for 4 h at 25°C. The electrolytic leakage (R1) was measured using a conductivity meter C700 (Eutech; Singapore) at 25°C. The samples tubes were then autoclaved for 15 min at 15 psi. The electrolytic leakage (R2) of the autoclaved samples was then measured at 25°C. The Relative electrolyte leakage was estimated using the formula (R1/R2) × 100.

#### Determination of the Malonyl Dialdehyde (MDA) Content

Lipid peroxidation was measured by thiobarbituric acid (TBA) test, which determines MDA as an end product of lipid peroxidation. Approximately, 100 mg of leaf material was homogenized in 1 ml 0.1% (w/v) TCA solution followed by centrifugation at 12,000 rpm for 15 min. After centrifugation 0.5 ml of the supernatant was collected to which 1 ml 0.5% (w/v) TBA in 20% TCA was added. The whole mixture was incubated at 95°C for 1 h and then incubated on ice for 5 min. The absorption (A_532_) was recorded after centrifugation at 10,000 rpm for 10 min. The value of non-specific absorption at A_600_ was subtracted. The MDA content was calculated from the extinction coefficient 155 mM^-1^ cm^-1^.

#### Measurements of Water Loss

The second upper leaf from all the samples was excised and dehydrated at room temperature by placing them on a blotting paper. The weight of the excised leaves was determined at definite time intervals (0, 2, and 4 h). Water loss was represented as the percentage of initial fresh weight at different time point.

### Expression and Purification of SbDHN1 Protein in Bacterial System

In order to express the *SbDhn1* gene in a heterologous host system, the coding sequence for *SbDhn1* was subcloned into the pET19b expression vector at *Nde*I and *Xho*I site (Supplementary Figure S2A). As the *SbDhn1* sequence comprises of a number of rare codons as shown in Supplementary Table S2; the expression of *SbDhn1* gene was achieved by transforming the construct into bacterial host strain, *Escherichia coli* Rosetta (DE3) pLysS. A culture of *E. coli* was grown at mid-log phase and induced with 1 mM Isopropyl β-D-thiogalactopyranoside (IPTG) for 8 h. The cells were pelleted and suspended in sonication buffer containing 20 mM Tris-HCl, pH 7.5, 1 mM EDTA, 1% TritonX100, 10 mM β-ME and 2 mM PMSF and sonicated on ice. The supernatant was separated by centrifugation at 10000 rpm for 10 min at 4°C and analyzed in a 15% SDS–PAGE.

SbDHN1 protein with N-terminal His-tag was purified by affinity chromatography according to manufacturer’s protocol. Briefly, Ni-NTA agarose column (Qiagen, Germany) was equilibrated with the binding buffer containing 50 mM NaH_2_PO_4_, 500 mM NaCl, and 10 mM imidazole and then incubated for 1 h at 200 rpm at 4°C with the induced supernatant. The column was washed with 50 mM NaH_2_PO_4_, 300 mM NaCl, and 20 mM imidazole. Finally, the protein was eluted with elution buffer containing 50 mM NaH_2_PO_4_, 300 mM NaCl, and 250 mM imidazole. Three fractions 1 ml each were collected and were checked in 15% SDS–PAGE. Purified protein sample from all the three elutes were loaded in 15% SDS–PAGE and blotted onto PVDF membrane. The membrane was blocked with BSA and after washing the membrane was probed using anti-His primary antibody (GeNie, India) (1:3000 dilution) and goat anti-rabbit HRP conjugated secondary antibody (GeNie, India) (1:3000 dilution). The immunoblotting procedure was conducted as described previously.

SbDHN1 protein sequence was analyzed by PONDR^2^ which predicts the disordered regions in the protein (Romero et al., 2001).

### Lactate Dehydrogenase Protection Assay

The LDH protection assay was performed to determine the protective effect of the SbDHN1 protein. The activity of LDH was measured by the modified technique of Lin and Thomashow (1992). Briefly, 0.03 U of LDH (Calbiochem, United States) was incubated at 54°C for 10 min in the presence or absence of the SbDHN1 protein (at a concentration of 50 ng/μl). Dehydration stress was imparted to LDH by vacuum drying the sample in presence or absence of SbDHN1 protein. After drying, the samples were rehydrated in 50 μl of 10 mM sodium phosphate buffer (pH 7.5). LDH activity was measured by diluting the enzyme mixture to 1 ml of reaction mix containing 1.1 mM pyruvic acid and 0.13 mM NADH. Oxidation of NADH was determined from the reading obtained at A_340_ for 5 min in a spectrophotometer (Beckman coulter, DU30) during the linear reaction rate. Known protectants like glycerol and BSA were used as positive controls in these experiments.

### Proteome Protection Assay

Total protein was isolated from 2-month-old sorghum plant. Briefly, 10 g of leaf tissue was homogenized in 50 ml protein isolation buffer (50 mM Tris-HCl, 100 mM NaCl, 1 mM DTT, 15 mM EGTA, 0.2% Triton X-100, 10% glycerol and 1 mM PMSF) in ice. After centrifugation at 3000 rpm for 10 min at 4°C; the supernatant was collected in a fresh tube and centrifuged at 10000 rpm for 10 min at 4°C. The clear supernatant was collected and dialyzed overnight in 10 mM sodium phosphate buffer (pH 7.5) at 4°C and centrifuged at 12,000 rpm for 10 min at 4°C before use. Protein concentration was determined spectrophotometrically (A_280_).

The dialyzed protein sample was incubated at 50°C in the presence or absence of equal amount of SbDHN1 protein for 10 min. The reaction mixture was centrifuged at 13,000 rpm for 20 min at 4°C. The supernatant and the pellet fraction was separated and analyzed in SDS–PAGE after dissolving the pellet in 10 mM sodium phosphate buffer (pH 7.5). The activity of the SbDHN1 protein was compared with other known protectants like glycerol and BSA.

The isolated protein from sorghum leaves was dried under vacuum in presence or absence of equal amount of SbDHN1 protein. The samples were then rehydrated in 50 μl of 10 mM sodium phosphate buffer (pH 7.5) and centrifuged at 13,000 rpm for 20 min at 4°C. The supernatant and the pellet fraction was separated and analyzed in SDS–PAGE. In order to serve as a control for these experiments, isolated sorghum leaf protein without any stress treatment was centrifuged at 13.000 rpm for 20 min at 4°C. The supernatant and pellet fraction was analyzed in SDS–PAGE as mentioned earlier.

### *In Vitro* Aggregation Assay

For *in vitro* aggregation assay LDH (from rabbit muscle cells) protein was incubated at 60°C for 0, 10, and 20 min in presence and absence of 50 μg SbDHN1 protein. The initial time point of 0 min served as a control for each experiment. The protein samples were stained with 150 mM Congo Red solution and observed under fluorescence microscope (Leica, Germany) under red filter with 40X objective.

### Isolation of Aggresomes

Aggresomes were isolated from stress-treated plants. Wild-type, empty vector transformed and *SbDhn1* transformed tobacco lines were subjected to high-temperature stress at 48°C for 4 h. For isolation of the aggresome; 100 mg of root tissue from wild-type, empty vector transformed and SbDhn1 transformed tobacco plants (lines 1, 6, and 10) was ground in 1 ml aggresome isolation buffer containing 50 mM HEPES pH 7.5, 150 mM NaCl, and 1% Triton X-100. The crushed samples were centrifuged at 1000 × *g* for 2 min in order to precipitate the debris. The supernatant containing the aggresomes were collected carefully without disturbing the samples. The collected supernatant was stained with 150 mM Congo Red solution and images were acquired in fluorescence microscopy (Leica, Germany) under the red filter with 40X objective.

### *In Vivo* Protein Aggregation Assay

*In vivo* protein aggregation assay was carried out with wild-type, empty vector transformed and *SbDhn1* transformed tobacco lines. The plants were subjected to high-temperature stress at 48°C for 4 h for the formation of aggregates. The leaf peels were isolated from third fully expanded leaf from each plant and stained with 150 mM Congo Red solution for 10 min. The leaf peels were then rinsed with deionized water and the peeled sections were observed under a confocal microscope (Olympus IX81).

## Results

### Isolation and Sequence Analysis of SbDhn1

**FIGURE 1:**
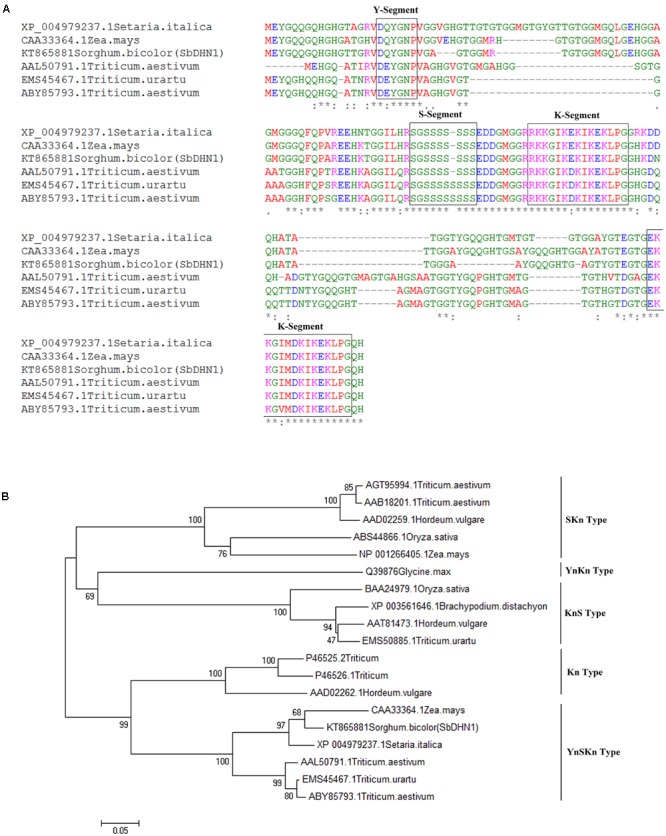
Sequence analysis of dehydrin **(A)** sequence alignment of SbDHN1 protein with other dehydrins from Poaceae **(B)** phylogenetic tree was drawn using the Neighbor-Joining method in MEGA6 where SbDHN1 and other dehydrins from Poaceae were used in the data set. Bootstrap value was obtained from 1000 replicates.

The PCR amplification of SbDhn1 coding sequence resulted in a 456 bp amplicon (Supplementary Figure S1A). The nucleotide sequence was deposited in GenBank with accession number KT865881. The multiple sequence alignment showed that the protein contained all the conserved sequence, characteristic of dehydrin (**Figure 1A**). SbDHN1 protein can be grouped as Y_n_SK_n_ type based on the phylogenetic analysis (**Figure 1B**). The SbDHN1 protein contained one Y-segment (18–23 residues), a stretch of serine residues S-segment (72–78 residues) and two lysine-rich K-segments at position (residues 88–102) and at the C-terminal end of the protein (residues 137–151) (Supplementary Figures S1B,C). *In silico* analysis of the protein sequence using ExPASy ProtParam calculated the isoelectric point to be 8.81, the molecular mass of 15.4 kD and grand average of hydropathicity (GRAVY)-1.132. All the physicochemical parameters of SbDHN1 protein, determined by ExPASy ProtParam tool, are shown in Supplementary Table S3.

### Real-Time Expression Analysis of SbDhn1 Gene under High Temperature and Osmotic Stress

**FIGURE 2:**
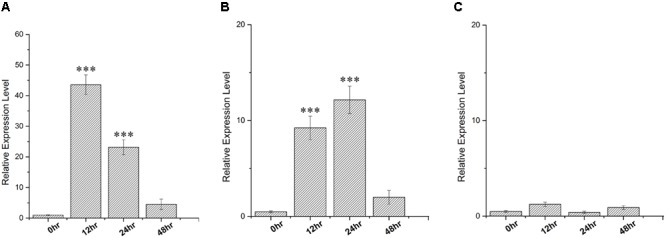
qRT PCR analysis carried out in *Sorghum bicolor* for relative expression of *SbDhn1* under different stress conditions at different time points (0, 12, 24, and 48 h) where the EIF1 gene was used as an internal control to normalize the expression level. **(A)** High temperature (48°C), **(B)** osmotic stress generated by supplementing 200 mM sorbitol in MS medium, and **(C)** low temperature (4°C). Expression levels were statistically analyzed against unstressed plants using student’s *t*-test (^∗∗∗^ *P* < 0.0001). Data shown are illustrative of at least three independent experiments.

In order to gain insight about the expression profile of the *SbDhn1* gene, qRT PCR analysis was carried out from 10 days old sorghum seedlings grown under different stress conditions. A time course experiment was carried out to determine the expression fold change in different time points. The expression analysis clearly indicates that the *SbDhn1* gene might be induced under high temperature. The expression of *SbDhn1* was found to be up-regulated during high temperature and osmotic stress condition (**Figures 2A,B**). Under high-temperature stress condition, the *SbDhn1* gene showed 44-fold upregulation at 12 h time point followed by a gradual decrease in expression of the *SbDhn1* gene. In contrast, upon imparting osmotic stress the *SbDhn1* gene showed ninefold upregulation after 12 h time point which reaches maxima of 12-fold after 24 h. After 48 h of osmotic stress, the expression level of *SbDhn1* gene decreased eventually. The expression level of *SbDhn1* gene in cold stress-treated plants showed no significant change among the different time points studied (**Figure 2C**).

### Analysis of Transgenic Tobacco Plants

Functional characterization of the protein was carried out by overexpressing the SbDHN1 protein in tobacco plants. The dehydrin gene was cloned in plant expression vector pGWB14 under the control of the CaMV35S promoter (Supplementary Figure S5). The transgenic lines were confirmed by PCR analysis using both *SbDhn1* and *hpt* gene-specific primers (Supplementary Figures S6A,B). Three PCR positive lines (Lines 1, 6, and 10) were randomly selected for transcript and western blot analysis. In reverse transcriptase PCR, all the three lines showed amplification of *SbDhn1* and *hpt* genes. In contrast, no amplification was visible in case of wild-type and empty vector transformed lines for *SbDhn1* gene, however, amplification of *hpt* gene was obtained for the empty vector transformed plants (Supplementary Figures S6C,D). The immunoblot analysis of the transgenic plants was carried out with an anti-HA antibody. The transgenic plants showed immunoreactive bands for the HA-tagged SbDHN1 protein. However, no band was detected in wild-type and empty vector transformed lines (Supplementary Figure S6E).

### Transgenic Tobacco Plants Overexpressing *SbDhn1* Gene Showed Better Growth under High Temperature and Osmotic Stress

**FIGURE 3:**
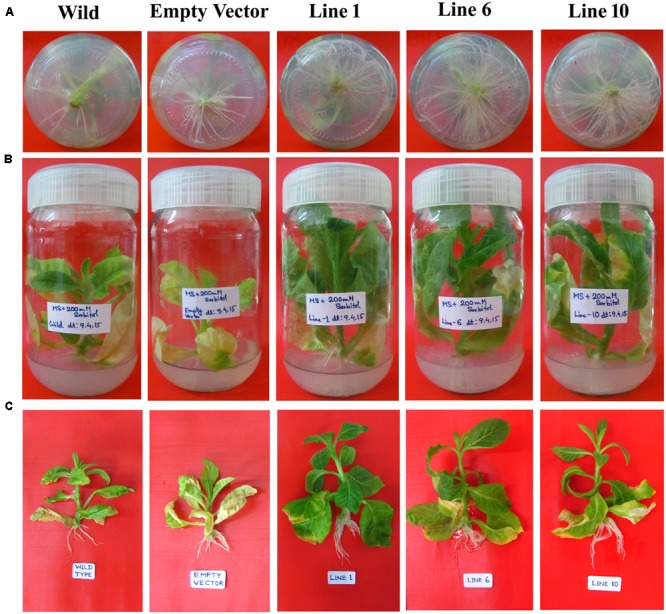
The *SbDhn1* transgenic lines along with wild and empty vector transformed plants grown in MS medium supplemented with 200 mM sorbitol. **(A)** Root growth of the plants after 21 days; **(B)** *SbDhn1* transgenic lines showing better growth compared to wild-type and empty vector transformed plants; **(C)** uprooted images of *SbDhn1* transformed tobacco lines along with wild-type and empty vector transformed plants.

**FIGURE 4:**
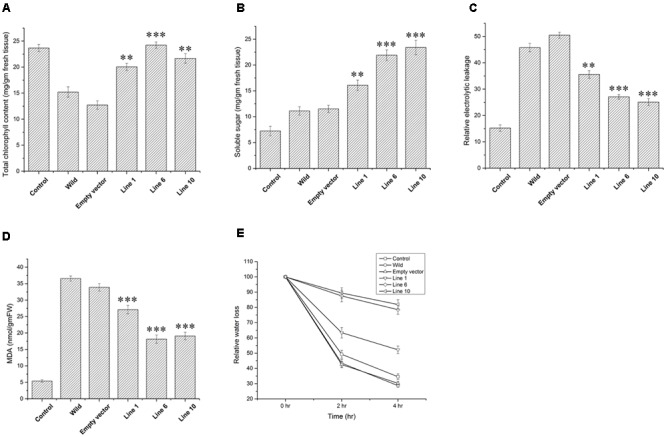
Measurements of different physiological parameters of *SbDhn1*transformed tobacco plants along with wild-type and empty vector transformed plants after 21 days of osmotic stress generated by supplementing 200 mM of sorbitol in MS medium. Unstressed wild-type plant served as a control in this experiment. **(A)** Total chlorophyll content **(B)** soluble sugar content **(C)** relative electrolytic leakage **(D)** malonyl dialdehyde (MDA) content **(E)** relative water loss. Error bar represents ± SEM from at least three experimental replicates. All data were statistically analyzed against unstressed plants using student’s *t*-test (^∗∗∗^ *P* < 0.0001, ^∗∗^ *P* < 0.001). Data shown are illustrative of at least three independent experiments.

Wild-type, empty vector transformed line and *SbDhn1* transformed tobacco plants were placed on MS medium supplemented with 200 mM sorbitol. The wild-type and empty vector transformed plants exhibited severe wilting, leaf yellowing and stunted growth compared to the transgenic plants. All the transgenic lines showed better growth under osmotic stress condition (**Figures 3A–C**). The transgenic lines also showed an increased amount of root growth compared to the wild or empty vector transformed lines (**Figure 3A**). The chlorophyll content and the soluble sugar content were found to be higher in transgenic plants compared to wild and empty vector transformed lines under stress condition (**Figures 4A,B**). To evaluate the cell membrane damage when the plants were subjected to osmotic stress, relative electrolytic leakage was measured. The wild-type plants kept under normal growth condition served as a control, which showed little electrolyte leakage. In contrast, the wild-type and the transgenic plants showed an increase in electrolyte leakage upon stress treatment. However, the electrolyte leakage was significantly higher in wild-type and empty vector transformed lines than transgenic lines (**Figure 4C**). The MDA content increased in all the plants when subjected to osmotic stress compared to the control plant. However, in the case of transgenic lines, the MDA content was significantly lower than wild-type and empty vector transformed lines (**Figure 4D**). The leaf water loss was significantly higher in wild-type and empty vector transformed lines with respect to the transgenic plants (**Figure 4E**).

**FIGURE 5:**
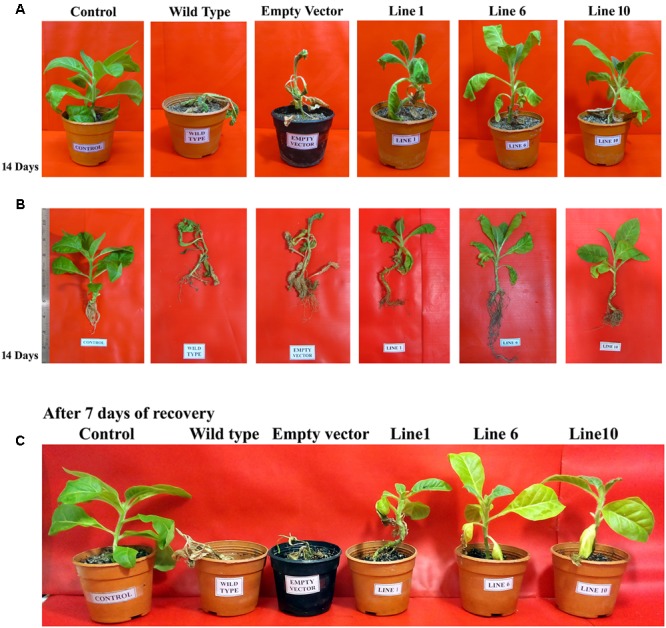
*SbDhn1* transformed tobacco lines along with wild-type and empty vector transformed plants were subjected to water stressed condition for 21 days along with 14 days of high temperature stress regime (as detailed in “Materials and Methods”). Unstressed wild-type plant served as a control in this experiment. **(A)** The transgenic plants showing uncompromised growth as compared to wild-type and empty vector transformed plants; **(B)** shoot and root growth conditions in uprooted images of plants; **(C)** transgenic plants showing better growth performance after 7 days of recovery as compared to the wild-type and empty vector transformed plants.

**FIGURE 6:**
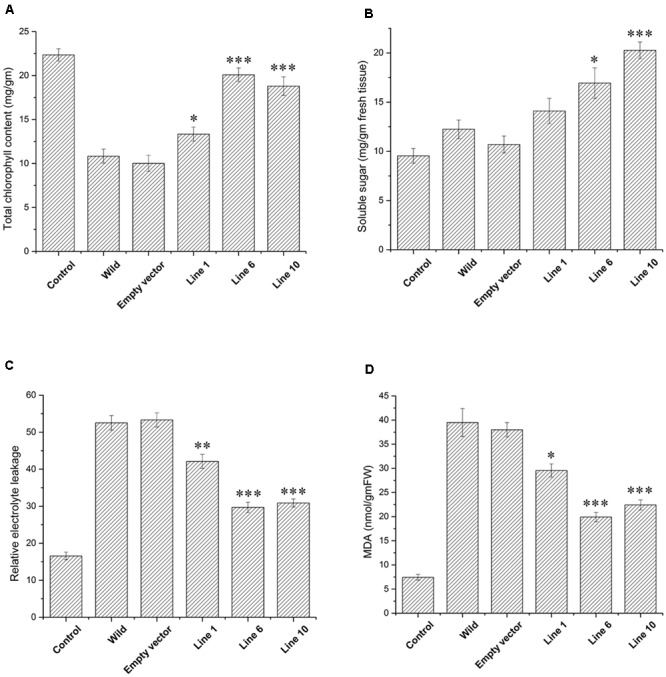
Measurements of different physiological parameters of *SbDhn1*transformed lines along with wild-type and empty vector transformed plants after 21 days of water stressed condition along with 14 days of high temperature stress regime. Unstressed wild-type plant served as a control in this experiment. **(A)** Total chlorophyll content **(B)** soluble sugar content **(C)** relative electrolytic leakage **(D)** MDA content. Error bar represents ± SEM from at least three experimental replicates. All data were statistically analyzed against unstressed plants using student’s *t*-test (^∗∗∗^ *P* < 0.0001, ^∗∗^ *P* < 0.001, and ^∗^ *P* < 0.01). Data shown are illustrative of at least three independent experiments.

The transgenic and empty vector transformed lines along with the wild-type plants were also assessed under simulating conditions of drought in soilrite by with-holding water for 7 days followed by a stress regime of combined high temperature and osmotic stress. After encountering 14 days of high temperature and osmotic stress condition simultaneously, wilting of the leaves were observed both in the transgenic, empty vector transformed lines and wild-type plants. However, the wilting was more pronounced in the case of wild-type and empty vector transformed plants (**Figure 5** and Supplementary Figure S9). The *SbDhn1* transgenic lines showed better growth as reflected by their fresh weight (data not shown). The chlorophyll and soluble sugar content also increased in *SbDhn1* transgenic lines (**Figures 6A,B**). The membrane integrity of the transgenic plants, empty vector transformed lines and wild-type plants along with the control plant were determined after stress treatment by measuring the electrolyte leakage. It was found that the relative electrolyte leakage increased in the plants after stress treatment. However, the electrolyte leakage was found to be lower in transgenic lines compared to the wild and empty vector transformed plants (**Figure 6C**). MDA content was found to increase both in the transgenic and wild-type plants after high temperature and osmotic stress for 14 days. However, the level of MDA in transgenic lines were significantly lower compared to the wild-type plant (**Figure 6D**). This suggests the overexpressing lines produced less amount of free radicals compared to the wild-type and empty vector transformed lines under stress condition. Experiments were carried to find out the degree of recovery of the plants after subjecting them to dehydration and high-temperature conditions. The transgenic lines recovered from stress conditions after watering them for 7 days under normal culture condition. The wild-type and empty vector transformed lines were not able to recover from the damaging stress situation (**Figure 5C**).

### Expression and Purification of SbDHN1 Protein

SbDHN1 protein was overexpressed in the bacterial host system in order to express and characterize the protein. Upon induction, a ∼20 kD protein product was expressed predominantly in the supernatant fraction as observed in SDS–PAGE (Supplementary Figure S2B). The high hydrophilicity, high percentage of glycine residues (27.6%) and the intrinsically disordered nature of the protein, (where 123 residues out of 152 are disordered, with overall disorderness of 80.92% as shown in Supplementary Figure S3) might account for the solubility and heat stability of the protein. The protein found to remain in the soluble condition even after boiling for 30 min (Supplementary Figure S2C). Protein with more than 95% homogeneity was obtained after Ni-NTA affinity purification (Supplementary Figure S2D) and confirmed by immunoblotting with an anti-His antibody (Supplementary Figure S2E).

### SbDHN1 Was Able to Protect the Enzyme Lactate Dehydrogenase under High Temperature and Osmotic Stress

**FIGURE 7:**
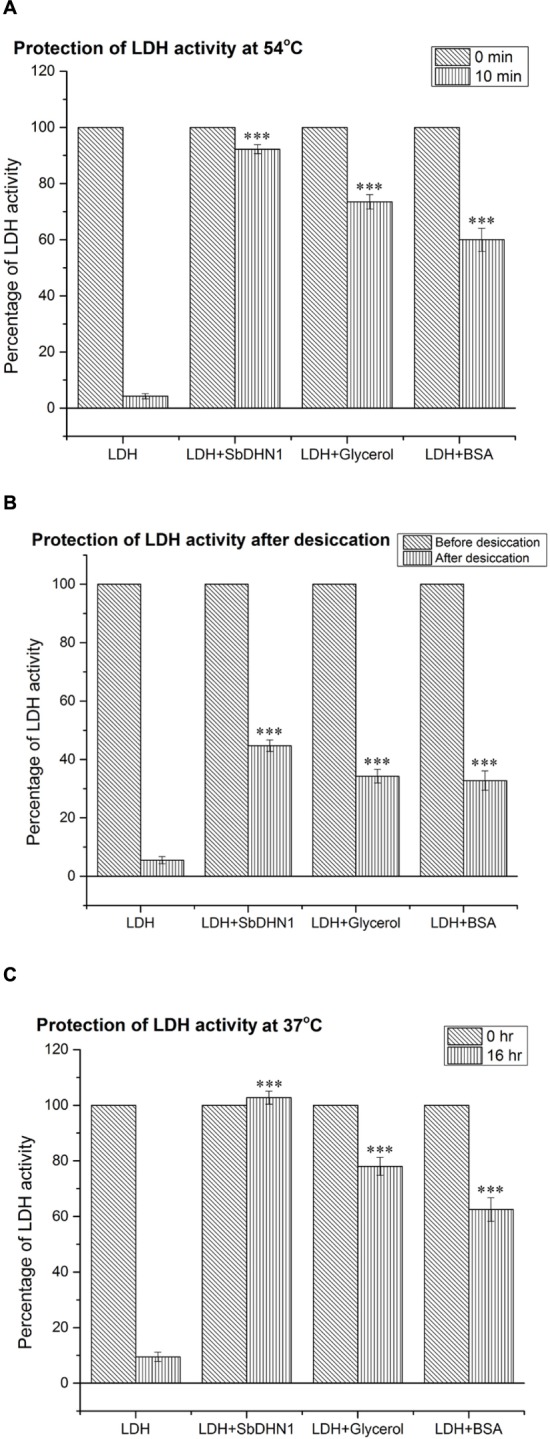
Lactate dehydrogenase activity by SbDHN1 protein under different stress conditions. **(A)** After heat shock treatment at 54°C for 0 and 10 min, in presence and absence of SbDHN1 protein. Glycerol and BSA were used as a positive control in this experiment; **(B)** lactate dehydrogenase (LDH) activity measured before and after desiccation treatment in presence and absence of SbDHN1 protein; the results were compared with that of glycerol and BSA **(C)** at 37°C after 0 and 16 h in presence and absence of SbDHN1 protein; the results were compared with that of glycerol and BSA. Error bar represents ± SEM from at least five replicates. All data were statistically analyzed against unstressed plants using student’s *t*-test (^∗∗∗^*P* < 0.0001). Data shown are illustrative of at least three independent experiments.

The enzyme LDH loses its activity completely upon incubation at 54°C for 10 min or at 37°C for 16 h. However, in the presence of 50 ng/μl SbDHN1 approximately 90% activity was found to be retained by the enzyme (**Figure 7A**). The enzyme activity was more than 100% in presence of SbDHN1 protein when incubated at 37°C (**Figure 7B**). A minimum concentration of 30 ng/μl was found to sufficient to retain 50% of the enzymatic activity under high-temperature stress (Supplementary Figure S4). Furthermore, we studied the effect of osmotic stress by vacuum drying the enzyme and then rehydrating the sample. The enzyme activity was studied in presence or absence of the SbDhn1 protein. The result shows that the enzyme LDH loses its activity upon vacuum drying, however, in presence of SbDHN1 approximately 50% of its activity was retained (**Figure 7C**). We have compared the activity of the SbDHN1 protein with other known protectants like glycerol and BSA. Our results showed that SbDHN1 protein could protect the LDH activity better than glycerol and BSA when subjected to high temperature or osmotic stress.

### Heat-Induced Aggregation of Protein Was Inhibited in Presence of SbDHN1 Protein under *In Vitro* Conditions

**FIGURE 8:**
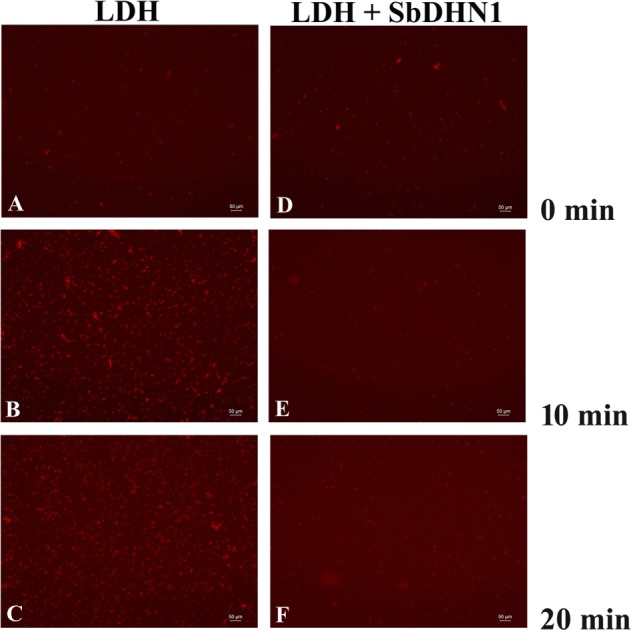
Lactate dehydrogenase was subjected to high temperature (60°C) in presence and absence of SbDHN1 protein in a time dependent manner (0, 10, and 20 min). Fluorescence microscope images were obtained after staining with Congo Red solution. **(A–C)** LDH in absence of SbDHN1 protein at 0, 10, and 20 min, respectively. **(D–F)** LDH in presence of SbDHN1 protein at 0, 10, and 20 min, respectively.

In order to elucidate the role of SbDHN1 protein in preventing the heat-induced aggregate formation, LDH was used as a model substrate which losses its activity at 54°C. LDH is a tetrameric enzyme that gets inactivated and forms aggregates at temperatures of 60°C or above which was observed by staining with Congo Red based on the phenomenon that protein aggregates can be visualized under a fluorescence microscope using Congo Red stain (Clement and Truong, 2014). Previously heat-induced protein aggregation of malate dehydrogenase (MDH) has been determined using Congo Red solution (Goloubinoff et al., 1999). Acquired images from fluorescence microscope clearly demonstrate that LDH forms irreversible aggregates when incubated at a high temperature of 60°C or above within minutes after incubation and reaches a maximum after approximately 20 min of incubation. This heat-induced aggregation of LDH was found to decrease by incubation with SbDHN1 protein in a concentration-dependent manner. The amount of aggregate formation increased with longer incubation period at high temperature. Increased amount of aggregate formation was observed when LDH was incubated at 60°C for 20 min, however, in presence of SbDHN1 protein the amount was significantly lower (**Figure 8**).

### In Presence of SbDHN1 Protein the Whole Proteome Remains Protected When Subjected to High Temperature or Osmotic Stress

**FIGURE 9:**
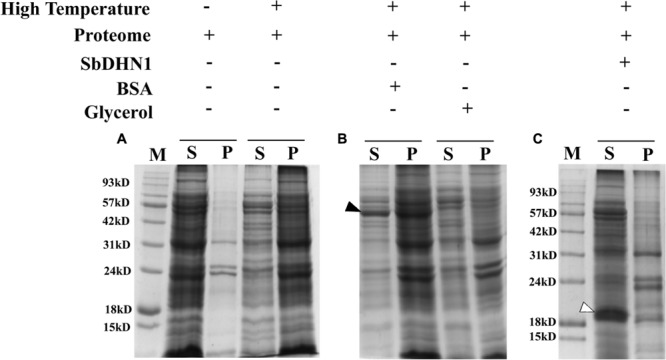
Proteome protection assay. **(A)** Figure represents the leaf proteome in presence and absence of high temperature (50°C); **(B)** leaf proteome in presence of BSA (indicated by a bold arrow) and Glycerol and **(C)** leaf proteome in presence of SbDHN1 (indicated by an open arrow). ‘S’ and ‘P’ represent the supernatant and pellet fraction after centrifugation and ‘M’ represents the protein molecular weight marker.

The whole proteome was subjected to *in vitro* high-temperature condition. This led to significant heat-induced aggregation of the protein, as many of the soluble proteins aggregated upon incubation at high temperature. Centrifugation was carried out in order to separate the insoluble proteins from the soluble fraction. The soluble fraction of proteins in the supernatant and the pelleted fraction were analyzed by SDS–PAGE after high-temperature stress as shown in **Figure 9**. A comparative analysis of the pelleted protein fraction with the soluble fraction showed that most proteins tend to get insoluble upon imparting high-temperature stress but not all of the molecules aggregate. Our results clearly demonstrate that solubility decreases depending upon the time of exposure of the proteins toward high-temperature stress. *In vitro* experiments were also carried out to determine the quantitative amount of protein that forms aggregates upon subjecting the proteome toward high temperature. Experimental results demonstrate an increase in aggregate formation with longer exposure time toward high-temperature stress. However, the addition of SbDHN1 protein at an approximate amount of 5 μg to an equal amount of the whole proteome prevents the protein from going to insoluble fractions by approximately 65–75% (**Figure 9**). The temperature-induced change in solubility of the whole proteome was eventually, partially protected by SbDHN1 protein. SDS–PAGE results showed little protein in the pelleted fraction and this observation could be correlated by determining the amount of protein in the supernatant (Supplementary Figure S8). Other proteins like BSA do not prevent changes in solubility of the whole proteome. Glycerol, a known osmoprotectant renders some degree of protection to the whole proteome from the heat-induced changes in solubility (**Figure 9**). SbDHN1 being an intrinsically disordered heat stable protein remained in the supernatant fraction. We even investigated the effect of the addition of SbDHN1 after temperature stress. Our experimental results demonstrate that addition of equal amount of SbDHN1 protein to the whole proteome after stress treatment have almost no protective effect as compared to its addition before the stress treatment.

We extended this analysis for the osmotic stress induced changes in solubility of the whole proteome by vacuum drying. Protein solubility decreased upon exposure to osmotic stress as resolved in the SDS–PAGE. However, supplementing SbDHN1 protein to the whole proteome before application of osmotic stress resulted in protection of the whole proteome (Data not shown).

### Anti-aggregation Role of SbDHN1 Protein in Living Plant Cells under High Temperature Condition

**FIGURE 10:**
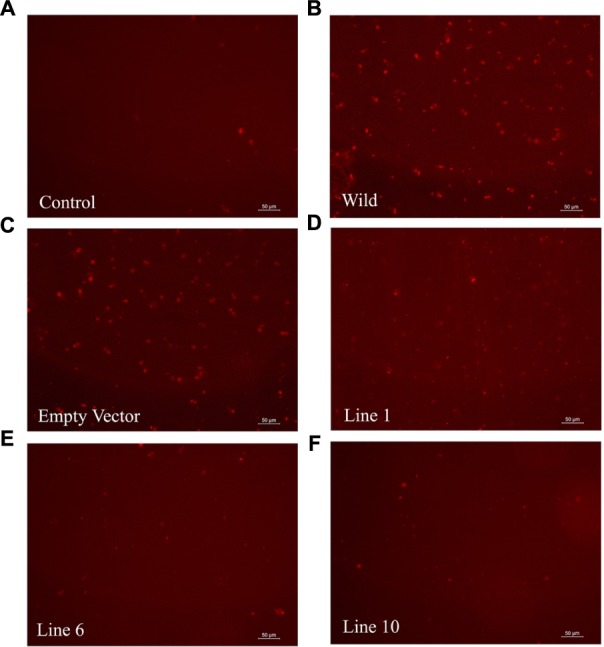
Florescence microscope images of isolated aggresomes from tobacco plants stained with Congo Red solution. **(A)** Wild-type tobacco plants grown under normal growth condition **(B)** wild-type tobacco plants kept at high temperature (48°C; 4 h), **(C)** empty vector transformed plant at high temperature **(D–F)** *SbDhn1* transformed transgenic lines (Lines 1, 6, and10, respectively) kept at high temperature.

**FIGURE 11:**
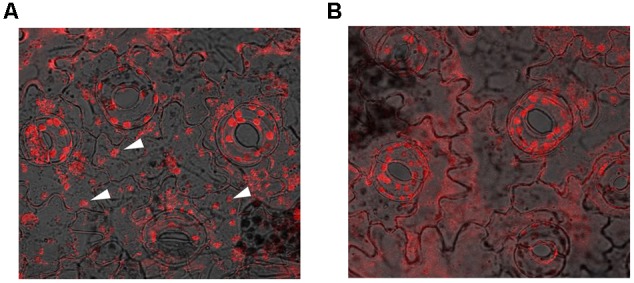
Confocal microscopic image of leaf peel sections of tobacco plants kept at high temperature (48°C; 4 h) and stained with Congo Red solution. **(A)** *In vivo* aggresome formation in wild-type tobacco plants (indicated by white arrows); **(B)** SbDhn1 transgenic lines showing reduced aggregates formation.

The ability of the SbDHN1 protein to protect a temperature-induced aggregation of the whole proteome in the hydrated state put forward one important question about whether this protein performs a similar function in living plant cells. To this end, we investigated the formation of aggregates in overexpressing lines of *SbDhn1* (lines 1, 6, and 10) and compared to that formed in the wild-type or empty vector transformed plants when subjected to high temperature for 4 h. Aggregates were isolated separately from all the lines as well as the wild-type and empty vector transformed plants. The isolated aggregates were stained with Congo Red and observed under fluorescence microscope. The untreated wild-type tobacco plants (control) showed almost no aggregate formation in concurrence with the fact that under normal growth condition the proteins do not encounter major conformational changes and remains in its active conformation. The aggregates isolated from wild-type and empty vector transformed plants showed a huge accumulation of aggregates when subjected to a stress condition. In contrast, upon subjecting the transgenic lines to high temperature fewer aggregates were observed as compared to the wild-type or empty vector transformed plants (**Figure 10**). However, the amount of aggregate in *SbDhn1* transformed plants (lines 6 and 10) were significantly lower in comparison to line 1. This might be presumably due to a high level of expression of SbDHN1 in lines 6 and 10. Among the three different lines chosen for expression analysis of HA-tagged SbDHN1 protein, line 6 showed the highest level of protein expression. Due to high expression level of SbDHN1 protein and low amount of aggregate formation, line 6 was chosen for further *in vivo* studies under a confocal microscope (Leica, Germany). The transgenic *SbDhn1* plant (line 6) along with wild-type tobacco plants were subjected to high temperature for 4 h. Leaf peel section of wild-type tobacco plants showed large protein aggregates in the cytoplasm as a result of protein aggregation under high-temperature stress condition when stained with Congo Red; whereas in the *SbDhn1* transformed (line 6) did not show any visible aggregates in the cytoplasm under confocal microscope studies (**Figures 11A,B**).

## Discussion

A condition of high temperature and osmotic stress is intimately associated with drought condition. In order to survive such harsh environmental conditions, plants have developed exclusive ways to ameliorate such stress assaults. Apart from the accumulation of osmoprotectants, compatible solutes and sugars, plants also synthesize a number of proteins to endure such stress conditions. Search programs had been undertaken in drought tolerant plants to enlist such proteins and validate their function *in planta*. Result analysis always supported the functional role of highly hydrophilic, intrinsically disordered LEA proteins during such stress conditions. Among the group of LEA proteins, dehydrins play a major role in protection during abiotic stress. Dehydrins are known stress-responsive proteins that are up-regulated in plants during stress such as drought, cold, and salinity (Hara et al., 2005). Phylogenetic analysis in conjunction with sequence analysis classified the dehydrin proteins into several groups. It had been proposed earlier that different group of dehydrins might perform different functions. Previous work from our laboratory also showed similar results (Agarwal et al., 2017). In agreement with our previous results here we provide direct evidence for the upregulation of *SbDhn1* gene during high temperature and osmotic stress compared to that in cold stress. This indicates that the primary role of SbDHN1 might be to impart protection to the plants during high-temperature stress and osmotic stress. The early upregulation of *SbDhn1* gene during high-temperature stress might be crucial in lending protection to the plants. To elucidate this idea we have ectopically overexpressed the gene in tobacco plants. The transgenic and wild-type plants when challenged with osmotic and (or) high-temperature stress showed interesting results. The data provide previously undescribed finding where we show that overexpressing *SbDhn1* lines rendered protection to the plants significantly when subjected to high temperature and osmotic stress. In our experiments, we have mimicked the environmental drought condition by imparting osmotic and high-temperature stress simultaneously as detailed in “Material and Methods.” As in the time of drought, the plants encounter both high temperature as well as osmotic stress due to unavailability of water. Earlier reports demonstrated limited protection of plants against high temperature upon overexpressing heat shock proteins. However, subjecting the plants to high temperature in overexpressing dehydrin plants had never been tested. There were no earlier reports on the protective effect rendered by overexpressing dehydrin at high temperature fitted in a diurnal pattern of stress treatment in transgenic plants. Therefore, this is the first report pointing toward the potentiality of dehydrin (SbDHN1) in rendering tolerance to the plants at high temperature and osmotic stress. Under normal growth conditions, there was no significant difference in plant morphology and different physiological parameters between the transgenic lines and the wild-type or empty vector transformed line. The transgenic plants showed significantly improved tolerance to high temperature and osmotic stress. Drought stress generally leads to the formation of secondary stress like oxidative stress due to increase in the ROS generation. Therefore the plants subjected to high-temperature stress frequently suffer from oxidative damage, leading to an increase in MDA content. However, the transgenic lines showed lower MDA content which might be due to the presence of a large number of glycine, histidine, and lysine residues in SbDHN1. Dehydrins can interact with membranes as evident from earlier reports (Danyluk et al., 1998; Nylander et al., 2001; Koag et al., 2003). The close interaction of dehydrin with membranes might result in stabilizing the membrane. This directly correlates well with the reduced relative electrolytic leakage as observed in the transgenic lines. The transgenic lines produced more chlorophyll and soluble sugar compared to the wild-type plants. The higher accumulation of the solutes provided the osmotic balance and membrane integrity. The relative water loss was significantly lower in transgenic lines justifying totally the protective role of dehydrin.

However, the levels of protection achieved in the overexpressed lines do vary significantly, tempting us to predict that increased level of the expressed protein might be the trick behind the dramatic performance under stress conditions. This might not be unrealistic as in order to achieve the molecular ‘shielding effect’ a high concentration of the protein remains mandatory. Our *in vitro* experiments justified this fact where a minimum concentration of the protein remains obligatory to render the protective effect to LDH. If we extend this justification for the whole proteome then definitely the high level of expression will be required for reflecting better performance. Under stress conditions, photosynthesis gets affected leading to reduced metabolites and energy. Under such a situation, the primary concern of the plants will be to increase the water uptake in one hand and minimize water loss on the other hand. The water uptake by the plants may be achieved by extending the primary roots as deep as possible. The extension of primary roots as an adaptive response might help in the better survival of the plants under drought condition. In this study, the transgenic lines exhibited better root architecture as compared to control plants. Root architecture observed in transgenic line (line 6) might be one reason for the better performance under stress situations. Therefore it is not unlikely that the transgenic plants were able to recover from damaging stress conditions accounting for all the factors discussed here.

After establishing that SbDHN1 was able to protect the plants under high temperature and osmotic stress, this study also describes the functional role of the protein in preventing high temperature-induced aggregation. Studies on protein function have shown the protective effect of dehydrin even better than known protectants like BSA or glycerol. In the previous report, citrus dehydrin was shown to be 20% more effective in rendering protection to MDH against desiccation, as compared to other known protectants like BSA (Sanchez-Ballesta et al., 2004). However, to the best of our knowledge, there is limited evidence for dehydrin exhibiting a protective role under high temperature and osmotic stress in conjunction. Only one group has addressed enzyme inactivation upon imparting high temperature where they have shown that wheat dehydrin could ameliorate the effect of high temperature under *in vitro* conditions. In presence of wheat dehydrin β-glucosidase and glucose oxidase could retain enzymatic activities when subjected to high temperature (Brini et al., 2010). In our earlier report SbDHN2, an SK_3_ type dehydrin from Sorghum was shown to protect LDH from high-temperature stress and cold stress (Halder et al., 2016). In agreement with our earlier report, a YSK_n_ type dehydrin from Sorghum (SbDHN1) rendered protection to the LDH enzyme under high temperature and osmotic stress.

Though it is possible to conclude the protective role of dehydrin under high temperature and osmotic stress but assigning a functional role of the protein in rendering such protections remained enigmatic. The previous report with another group of LEA proteins demonstrated prevention of aggregate formation under both *in vitro* and *in vivo* conditions (Chakrabortee et al., 2007). It might not be important whether aggregates are formed under *in vitro* condition as long as the protein remains in its active conformation. However, the formation of aggregates under *in vivo* conditions might have a damaging outlook for the cell. In this current study, we provide experimental evidence for heat-induced aggregate formation under high temperature for an enzymatic protein like LDH. Incubation at high temperature in presence of SbDHN1 protein, however, resulted in fewer aggregates. The binding of Congo Red to heat-denatured protein on the exposed hydrophobic surfaces was well documented in Goloubinoff et al. (1999). This demonstration is in line with the previous report which showed that LEA proteins were capable of protecting the cellular proteins or enzymes against drying and heat-induced aggregation (Goyal et al., 2005; Chakrabortee et al., 2007; Tunnacliffe and Wise, 2007; Tompa and Kovacs, 2010; Hughes and Graether, 2011; Hughes et al., 2013). Additionally, it was also seen SbDHN1 could not revert back an aggregated protein back to its natural conformation. Taken together, these results provide clearer evidence for the role of dehydrin under the high-temperature condition: being very effective at suppressing aggregation comparable to that of known osmoprotectant like glycerol. This by far remains a property not shared by all proteins. We further extended our study to find the non-discriminatory protective effects of SbDHN1; the whole proteome of sorghum plant was subjected to high-temperature stress in presence and absence of SbDHN1 protein. The Incubation of the whole leaf proteome at high temperature in presence of SbDHN1 protein, however, resulted in fewer aggregates.

*In vivo* analysis carried out in the overexpressed transgenic lines provided interesting datasets. Overexpressed SbDHN1 transgenic lines suppressed aggregate formation more as compared to wild-type. This suppression of aggregate formation remained proportional to the expression level of the SbDHN1 protein. Pending accumulation of more data, we can nevertheless begin to formulate a hypothesis about the role of the dehydrin under high temperature and osmotic stress. Under high temperature and osmotic stress, macromolecular crowding effect of dehydrin molecules might prevent the collapse of the protein molecules. The molecular crowding effect makes unfolding of protein molecules as energetically unfavorable. Furthermore, an intrinsically disordered structure of dehydrins allows them to act as a molecular shield preventing aggregation of neighboring protein molecules. The evidence provided here showed the protective role of dehydrin in protecting the whole leaf proteome partially under both high temperature and osmotic stress, indicated the possibility of a broad and a rather non-specific mechanism of action for SbDHN1 protein. The possible non-discriminatory protective effects of SbDHN1 might be due to high flexibility charged nature of the molecule limiting the interactions between neighboring molecules by providing a ‘shielding effect.’

## Author Contributions

All authors have participated sufficiently in the work to take public responsibility for appropriate portions of the content. No one, other than the authors listed below has contributed substantially to the writing and revising of the manuscript. Contributors who do not meet the criteria for authorship have been listed in the acknowledgment. Study conception and design: SR and TH. Acquisition of data: TH and GU. Analysis and interpretation of data: TH and GU. Drafting of manuscript: SR, TH, and GU. Critical revision: SR.

## Conflict of Interest Statement

The authors declare that the research was conducted in the absence of any commercial or financial relationships that could be construed as a potential conflict of interest.

## Footnotes

1 http://www.ebi.ac.uk/Tools/msa/clustalo/

2 http://www.pondr.com/
