# Supplementary material for: YSK2 Type Dehydrin (SbDhn1) from Sorghum bicolor Showed Improved Protection under High Temperature and Osmotic Stress Condition
Source: Front Plant Sci. 2017 May 30;8:918. doi: 10.3389/fpls.2017.00918 (PMC5447703; doi:10.3389/fpls.2017.00918)
Supplement: Supplementary file 1 [file Data_Sheet_1.PDF]

Supplementary Material

YSK$_2$ type dehydrin (*SbDhn1)* from *Sorghum bicolor* showed improved protection under high temperature and osmotic stress condition

Tanmoy Halder[1], Gouranga Upadhyaya[1], Sudipta Ray[1*]

[1]Plant Functional Genomics Laboratory, University of Calcutta, Department of Botany, Centre of Advanced Study, Kolkata, WB, India.

***Correspondence:** Dr. Sudipta Ray; E-mail: srbot@caluniv.ac.in

Supplementary Table S1: List of primers used.

Name of the gene	Sequence of the oligo(s) used
Dehydrin (*SbDhn1*)	5′ *CATATG*GAGTACGGTCAGCAGGGACAGCAC 3′
	5′ *CTCGAG*GTGCTGTCCGGGCAGCTTCTCCTTG 3′
Hygromycin (*hpt*)	5′ ATGAAAAAGCCTGAACTCACCGCGAC 3′
	5′ TTCCTTTGCCCTCGGACGAGTGCTG 3′
Realtime primers for *SbDhn1*	5′ GAAGGAGGAAGAAGGGAATCAAG 3′
	5′ GTGTGTTCTTGCTGCCCGTA 3′
Realtime primers for EIF1	5′ CACTCTTGGTGTCAGACAGAT 3′
	5′ ACCTTCTTGAGGTAGGAACC 3′

Supplementary Table S2: Rare codons present in *SbDhn1*

**Rare codons**	**Translated Amino acids**	**Number of occurrences**	**Occurring at codons**
AGG	Arg	4	32, 58, 86, 87
CCC	Pro	2	100, 149

Supplementary Table S3: Physicochemical properties of SbDHN1

Number of amino acids	152
Molecular weight	15426.8 Dalton
Theoretical pI	9.33
Total number of negatively charged residues	15
Total number of positively charged residues	19
Aliphatic index	32.17
Grand average of hydropathicity (GRAVY):	-1.138

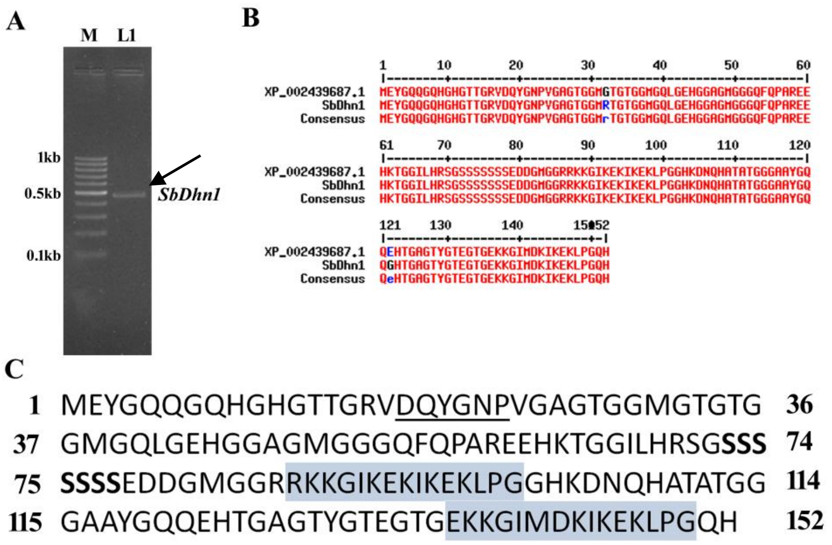

Supplementary Figure S1: PCR amplification and sequence analysis of dehydrin (*SbDhn1)* gene from *Sorghum bicolor.* **(A)** Agarose gel electrophoresis of the amplified PCR product of *SbDhn1* (L1) along with 100 bp DNA ladder (M). **(B)** Alignment of translated SbDHN1 sequence with reported sequence available in NCBI database **(C)** Amino acid sequence of the SbDHN1 protein. The S segments represented in bold and boxes representing the K segment(s).

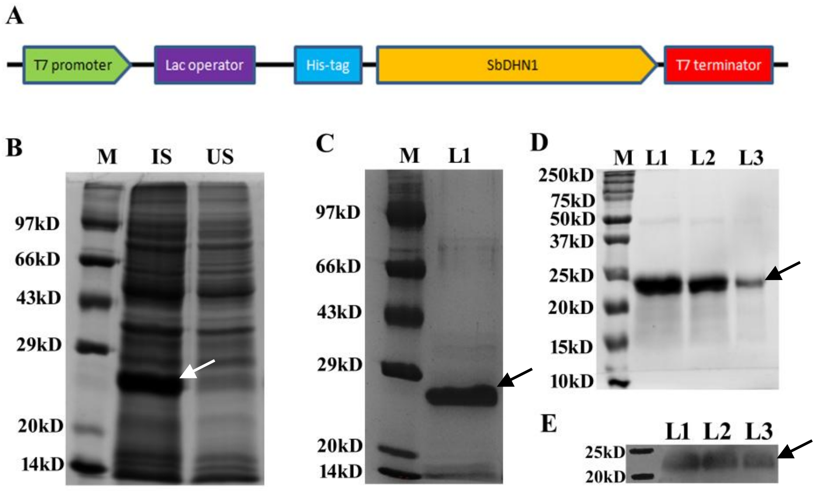

Supplementary Figure S2: Expression cassette, expression profile, purification and heat stability of the recombinant SbDHN1 protein. **(A)** Schematic representation of expression cassette **(B)** Coomassie stained SDS-PAGE showing the expression of SbDHN1 protein with His-tag. Protein marker (Lane M), induced supernatant (Lane IS), uninduced supernatant (Lane US), **(C)** SDS-PAGE stained with coomassie brilliant blue showing the presence of heat stable SbDHN1 protein (L1) and Protein marker (M). **(D)** Purification of SbDHN1 protein with Ni-NTA agarose (L1) and protein marker (M) **(E)** Immunoblot of purified SbDHN1 protein with anti-His antibody (L1 to L3).

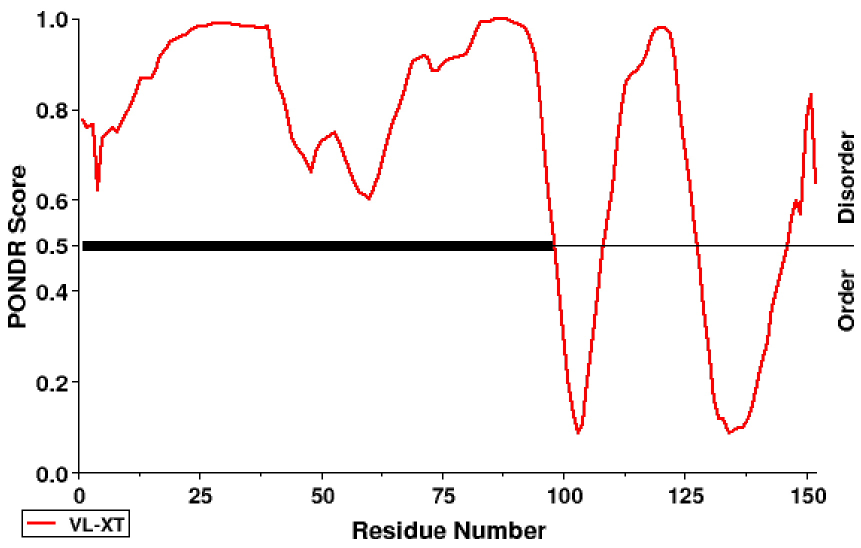

Supplementary Figure S3: PONDR output showing the distribution of disordered regions in the SbDHN1 protein sequence.

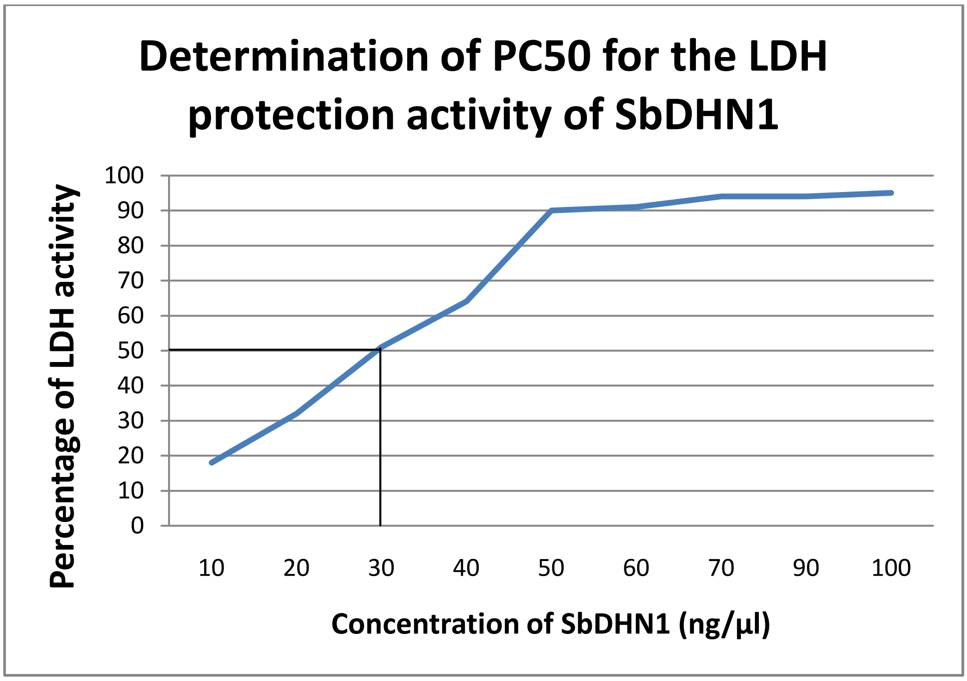

Supplementary Figure S4 : Determination of 50% Protective Concentration (PC50) for the LDH protection activity of SbDHN1

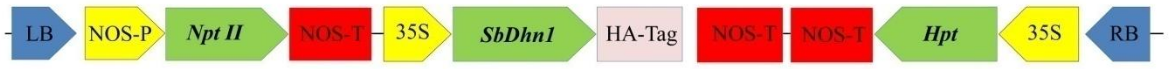

Supplementary Figure S5: Schematic representation of the plant expression cassette.

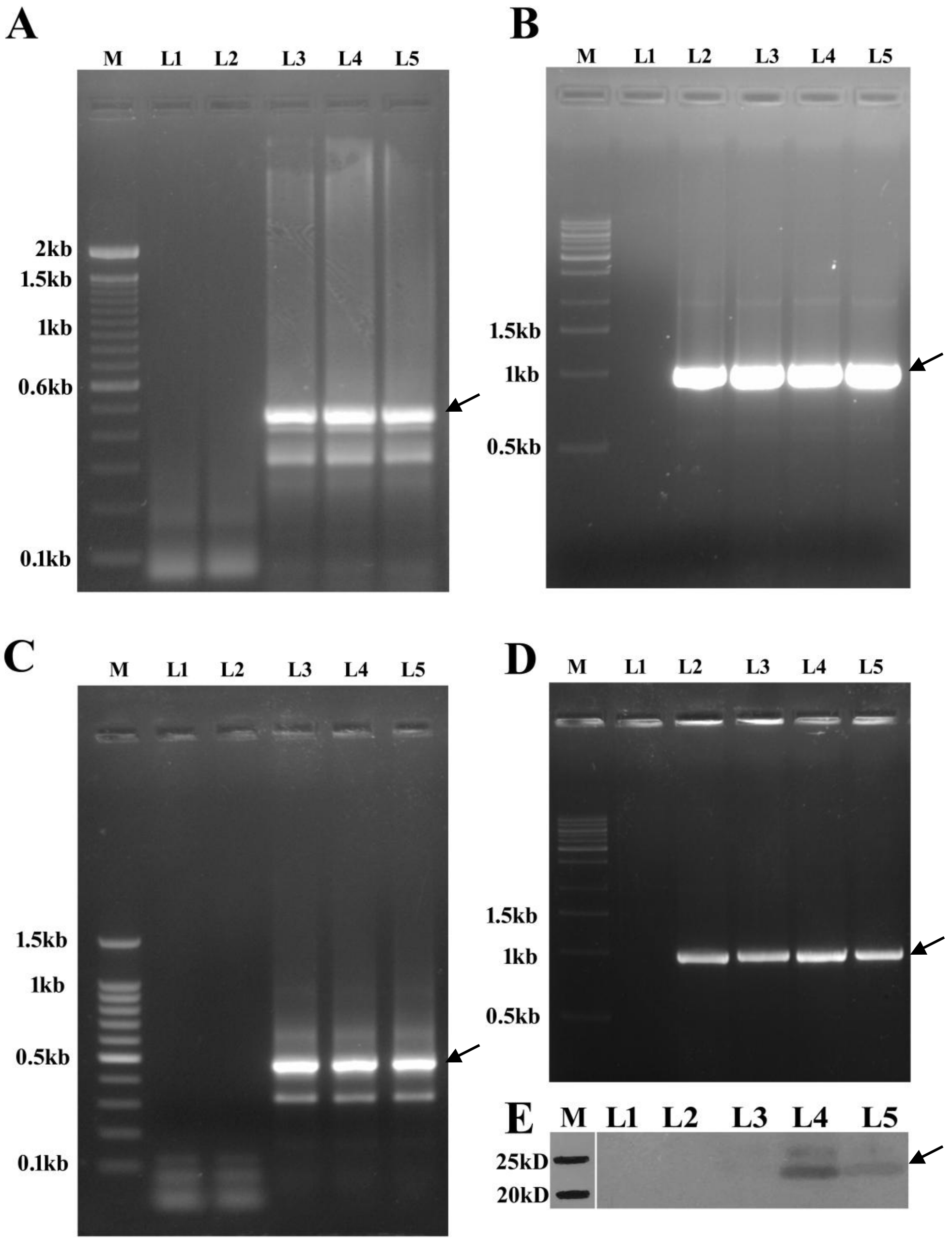

Supplementary Figure S6: Screening of *SbDhn1* transgenic plants. **(A)** PCR amplification of *SbDhn1* from genomic DNA from wild-type plant (L1), empty vector transformed line (L2), *SbDhn1* transgenic lines 1, 6 and 10 (L3, L4 and L5 respectively); **(B)** PCR amplification of *hpt* from genomic DNA from wild-type plant (L1), empty vector transformed line (L2), *SbDhn1* transgenic lines 1, 6 and 10 (L3, L4 and L5 respectively); **(C)** PCR amplification of *SbDhn1* from cDNA of wild-type plant (L1), empty vector transformed line (L2), *SbDhn1* transgenic lines 1, 6 and 10 (L3, L4 and L5 respectively); **(D)** PCR amplification of *hpt* from cDNA of wild type plant (L1), empty vector transformed line (L2), *SbDhn1* transgenic line 1, 6 and 10 (L3, L4 and L5 respectively); **(E)** Immunoblot analysis of wild-type plant (L1), empty vector transformed line (L2), *SbDhn1* transgenic lines 1, 6 and 10 (L3, L4 and L5 respectively).

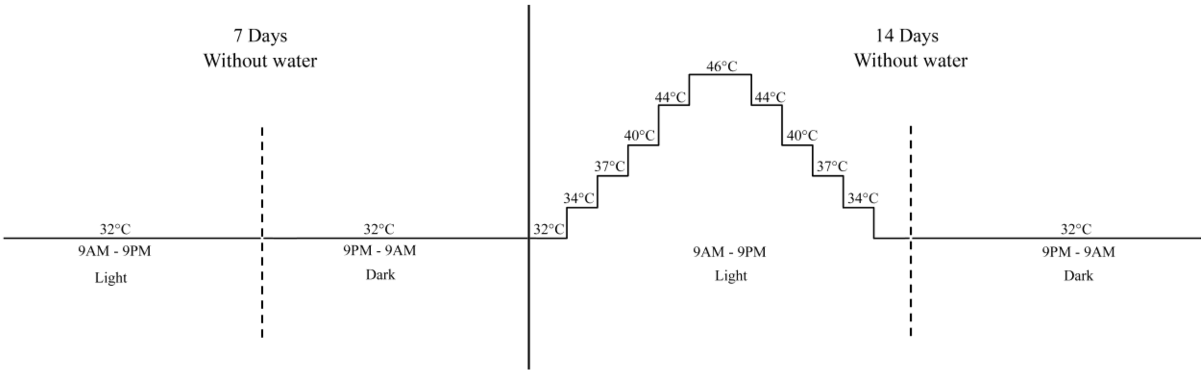

Supplementary Figure S7: Stress regime under which the wild-type plants, empty vector and *SbDhnI* transformed transgenic lines were kept.

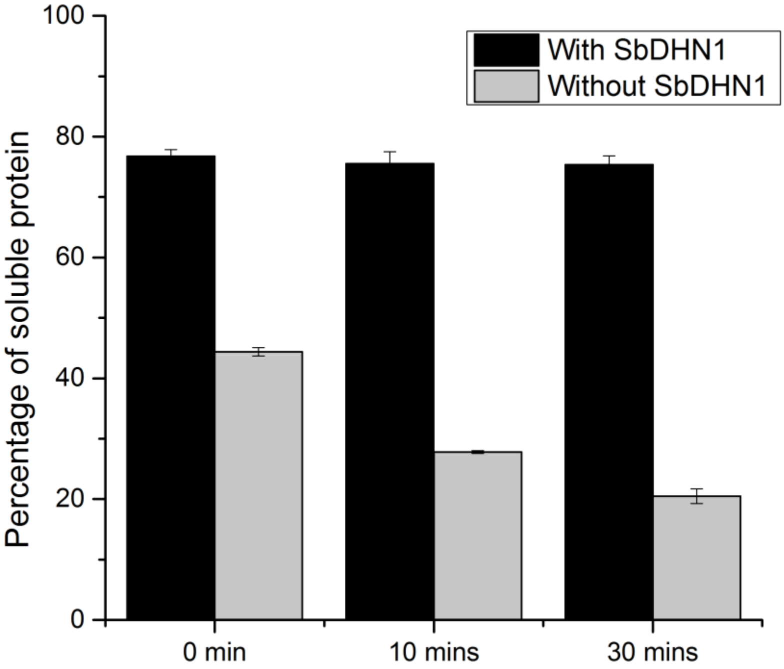

Supplementary Figure S8: Quantitative representation of the amount of protein in the supernatant after high temperature stress treatment of the leaf proteome in presence and absence of SbDHN1 protein at different time intervals.
