# Supplementary material for: YSK2 Type Dehydrin (SbDhn1) from Sorghum bicolor Showed Improved Protection under High Temperature and Osmotic Stress Condition
Source: Front Plant Sci. 2017 May 30;8:918. doi: 10.3389/fpls.2017.00918 (PMC5447703; doi:10.3389/fpls.2017.00918)
Supplement: Supplementary file 2 [file Data_Sheet_2.PDF]

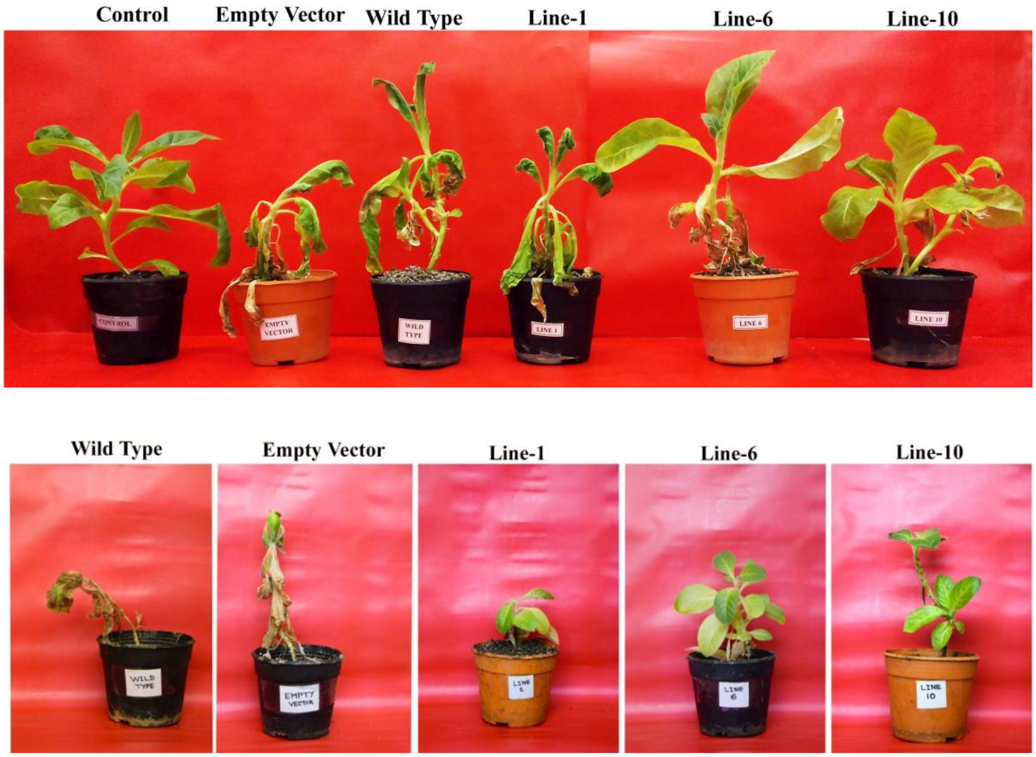

Supplementary Figure S9: *SbDhn1* transformed tobacco plants along with wild type, empty vector transformed after 14 days of high temperature and osmotic stress.
